# Evaluation of Polyurea-Crosslinked Alginate Aerogels for Seawater Decontamination

**DOI:** 10.3390/gels7010027

**Published:** 2021-03-04

**Authors:** Patrina Paraskevopoulou, Grigorios Raptopoulos, Faidra Leontaridou, Maria Papastergiou, Aikaterini Sakellari, Sotirios Karavoltsos

**Affiliations:** 1 Inorganic Chemistry Laboratory, Department of Chemistry, National and Kapodistrian University of Athens, Panepistimiopolis Zografou, 15771 Athens, Greece; grigorisrap@chem.uoa.gr (G.R.); faidraleo@chem.uoa.gr (F.L.); mapapast@chem.uoa.gr (M.P.); 2 Laboratory of Environmental Chemistry, Department of Chemistry, National and Kapodistrian University of Athens, Panepistimiopolis Zografou, 15784 Athens, Greece; esakel@chem.uoa.gr

**Keywords:** alginate aerogels, environmental remediation, polymer-crosslinked aerogels, seawater, water decontamination

## Abstract

Polyurea-crosslinked Ca-alginate (X-Ca-alginate) aerogel beads (diameter: 3.3 mm) were evaluated as adsorbents of metal ions, organic solvents, and oils. They were prepared via reaction of an aromatic triisocyanate (Desmodur RE) with pre-formed Ca-alginate wet gels and consisted of 54% polyurea and 2% calcium. X-Ca-alginate aerogels are hydrophobic nanoporous materials (90% v/v porosity), with a high BET surface area (459 m^2^/g^−1^), and adsorb Pb^II^ not only from ultrapure water (29 mg/g^−1^) but also from seawater (13 mg/g^−1^) with high selectivity. The adsorption mechanism involves replacement of Ca^II^ by Pb^II^ ions coordinated to the carboxylate groups of the alginate backbone. After treatment with a Na_2_EDTA solution, the beads can be reused, without significant loss of activity for at least two times. X-Ca-alginate aerogels can also uptake organic solvents and oil from seawater; the volume of the adsorbate can be as high as the total pore volume of the aerogel (6.0 mL/g^−1^), and the absorption is complete within seconds. X-Ca alginate aerogels are suitable for the decontamination of aquatic environments from a broader range of inorganic and organic pollutants.

## 1. Introduction

Water pollution is one of the most serious problems facing humanity, becoming even more severe over the last century because of industrialization. Pollutants can be roughly categorized as inorganic (e.g., metal species) and organic (e.g., solvents, dyes, pesticides), all posing a serious threat to human health. For example, lead causes encephalopathy, neuropathy, reduced intellectual capacity in children, and kidney damage [1]; cadmium is carcinogenic and causes kidney and skeletal damage [1]; dichloromethane shows toxicity for the liver and kidneys and is harmful to the nervous and reproductive systems [2]; and chloroform is carcinogenic and causes liver damage [3].

Several technologies have been developed for the capture, removal, and sometimes recovery of pollutants, including chemical/electrochemical and physical methods, such as chemical/electrochemical precipitation, membrane separation/electrolysis, liquid extraction, adsorption, coagulation, flotation, crystallization, and others [4,5,6,7]. All these methods have their pros and cons. In addition, adapting a method that was originally developed for decontamination of fresh water to decontamination of seawater can be very challenging because of the presence of metal cations and anions in seawater, which may interfere with the method in various ways [8,9,10,11].

Among the technologies developed for water decontamination, adsorption is one of the most preferable methods because it is easy to apply and cost-efficient, and because there is usually a wide range of materials that can be used as adsorbents for a specific pollutant or class of pollutants [12]. Such materials include carbon-based materials, organic polymers, polyelectrolytes, metal-organic frameworks (MOFs), sol-gel materials, and composites thereof [13,14,15,16,17,18,19,20,21,22,23,24,25,26]. Among them, biopolymer aerogels and composite materials derived from them have attracted considerable attention [10,11,15,23,27,28,29] because they combine the highly porous nanostructure of aerogels with the characteristic properties of biopolymers, which come from natural products and are biocompatible and biodegradable.

Expanding the polymer-crosslinked (X-aerogel) technology that was initially developed with silica [30,31,32,33], and other metal oxide aerogels [34,35,36,37,38], we recently introduced a new class of X-aerogels based on polyurea-crosslinked biopolymers (referred to as X-alginate and X-chitosan aerogels) [39,40,41]. X-aerogels are prepared from pre-formed wet-gel networks (of an inorganic oxide or a biopolymer) via reaction of the functional groups on the surface of their skeletal framework (e.g., –OH or –NH_2_) with a suitable monomer (e.g., a multifunctional isocyanate). The reaction leads to formation and accumulation of a nano-thin conformal polymer coating over the entire inorganic or biopolymer skeletal framework. The distinguishing feature of X-aerogels is their greatly enhanced mechanical strength compared to that of native aerogels, while other properties related to the nanoporous structure, e.g., high surface area, low thermal conductivity, low dielectric constant, and high acoustic attenuation, are affected only to a small extent. In addition, X-alginate aerogels are hydrophobic, while native alginate aerogels are extremely hydrophilic [39]. Use of different multifunctional isocyanates tunes the material properties from the chemical composition perspective [39,41]. Overall, the properties of X-alginate aerogels make them promising materials for several applications, including thermal and acoustic insulation and as adsorbers for environmental remediation.

**Scheme 1:**
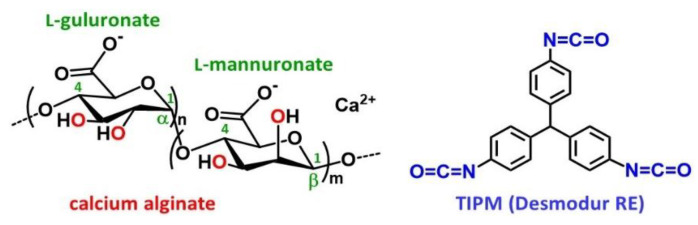
The structures of calcium alginate and of the isocyanate Desmodur RE (triphenylmethane-4,4’,4’’-triisocyanate (TIPM)).

In line with the above, in this paper, we report an initial evaluation of X-Ca-alginate aerogels prepared from Ca-alginate and an aromatic triisocyanate (Desmodur RE; Scheme 1) toward environmental remediation. This initial study focuses on the adsorption of lead and selected organic solvents and oil from seawater, taking advantage of the stability of X-Ca-alginate aerogels in seawater.

## 2. Results and Discussion

### 2.1. Preparation and Characterisation of X-Ca-Alginate Aerogel Beads

**Scheme 2:**
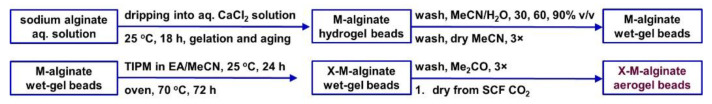
Synthesis of native calcium alginate and crosslinked Ca-alginate (X-Ca-alginate) wet-gel and aerogel beads.

**Scheme 3:**
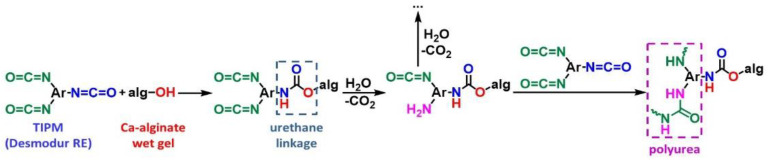
The reaction of crosslinking Ca-alginate wet gels with TIPM (Desmodur RE).

X-Ca-alginate aerogel beads were prepared as outlined in Scheme 2. The process starts with the synthesis of calcium alginate hydrogel beads according to literature procedures [38,39], via the dropwise addition of an aqueous sodium alginate solution into an aqueous solution of CaCl_2_, causing immediate gelation. After aging for 18 h, the beads were crosslinked with Desmodur RE (triphenylmethane-4,4’,4’’-triisocyanate (TIPM); Scheme 1) to produce the corresponding crosslinked aerogel beads (referred to as X-Ca-alginate aerogel beads; 3.3 mm in diameter), as described in Section 4. The chemical transformations occurring during crosslinking are summarized in Scheme 3. As described previously [39,40,41], the triisocyanate reacts with the –OH groups of the alginate, forming urethane linkages to the backbone polymer, and then the remaining –NCO groups are hydrolyzed to –NH_2_ groups by water remaining adsorbed onto the surface of the alginate network. The –NH_2_ groups react further with still free triisocyanate in the pores, forming urea groups and eventually polyurea. X-Ca-alginate wet-gel beads were solvent-exchanged with acetonitrile and were dried into aerogels with liquid CO_2_ taken out at the end as a supercritical fluid. An optical photograph, size distribution, and mean diameter of X-Μ-alginate aerogel beads are shown in Supplementary Materials Figure S1.

The chemical identity of the X-Ca-alginate aerogel beads was confirmed with attenuated total reflection Fourier transform IR (ATR-FTIR) (Supplementary Materials Figure S2), solid-state cross-polarization magic angle spinning (CPMAS) ^13^C NMR (Supplementary Materials Figure S3), and atomic absorption spectrometry. The ATR-FTIR and ^13^C CPMAS spectra showed characteristic peaks of both the alginate backbone and the polyurea (PUA) crosslinking polymer and agreed with those reported previously [41]. The calcium content of X-Ca-alginate aerogels was measured with atomic absorption spectroscopy, and it was found equal to 1.9% ± 0.2% *w*/*w* (0.047 mol per 100 g of aerogel). The PUA content, calculated from the skeletal density of X-Ca-alginate aerogel beads (Supplementary Materials Table S1) and the skeletal density of the corresponding native alginate aerogels (2.07 g/cm^−3^) [41], was found to be approximately 54% *w*/*w*.

Selected material properties of X-Ca-alginate aerogel beads are summarized in Supplementary Materials Table S1 and are in agreement with those reported previously [41]. In brief, the aerogels in this study have low bulk density (0.15 g/cm^−3^), high BET (Brunauer–Emmett–Teller) surface area (459 m^2^/g^−1^), and high porosity (90% v/v). The N_2_-sorption isotherm (Supplementary Materials Figure S4) showed a narrow loop and did not reach saturation, characteristic of macroporous materials with some mesoporosity. This conclusion is supported by the fact that *V*_Total_ (calculated from bulk and skeletal densities) is larger than *V*_1.7–300nm_ (from N_2_-sorption). Some microporosity (6% of the BET surface area, 1.8% of *V*_Total_), as indicated by the early rise of the isotherm at low partial pressures, is attributed to the rigid aromatic core of TIPM [42,43,44,45,46,47,48,49]. The BJH (Barrett–Joyner–Halenda) curves (Supplementary Materials Figure S4), for pores in the range of 1.7–300 nm, were broad, as expected for networks formed after particle aggregation and showed a maximum at 44 nm.

### 2.2. Adsorption of Pb^II^ from Water

**Figure 1:**
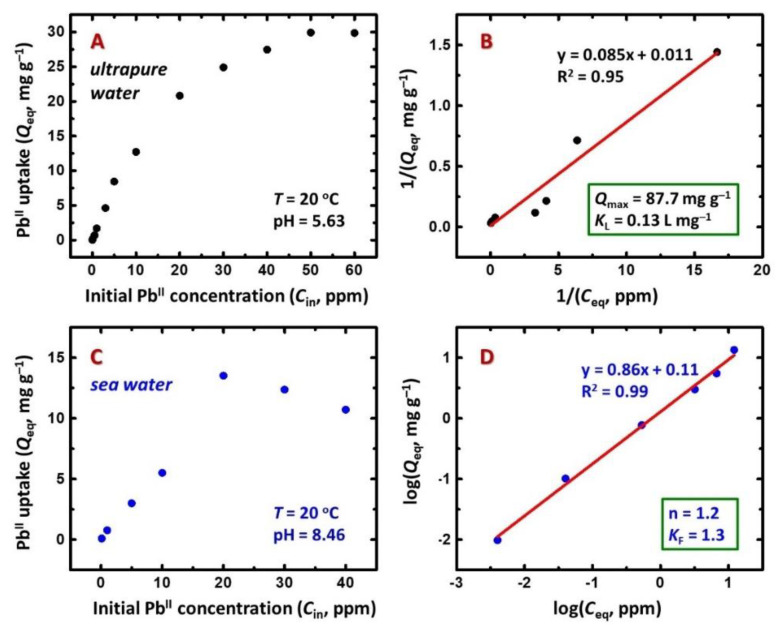
Isotherms for Pb^II^ uptake from ultrapure water (top row) and seawater (bottom row) solutions by X-Ca-alginate aerogel beads. (**A**,**C**) Pb^II^ uptake at equilibrium (*Q*_eq_) versus the initial concentration of Pb^II^ (*C*_in_). (**B**) Langmuir isotherm. (**D**) Freundlich isotherm. *C*_eq_ is the concentration of Pb^II^ in the solution at equilibrium.

X-Ca-alginate aerogel beads were tested for the adsorption of heavy metal ions from aqueous solutions. First, the beads were kept in a solution containing 100 mg/L^−1^ of each of the following metal ions: Al^III^, As^III^, V^III^, Cr^III^, Mn^II^, Fe^III^, Co^II^, Ni^II^, Cu^II^, Zn^II^, Cd^II^, Ru^II^, Sr^II^, Cs^I^, Ba^II^, and Pb^II^. Determination of the equilibrium concentration of each metal ion showed that Pb^II^ was selectively adsorbed from the above solution, in agreement with the expected relative affinity of alginates to metal ions, as has been reported in the literature [50]. Therefore, the uptake of Pb^II^ was studied more thoroughly, using standard solutions of various concentrations in the range of 0.01 to 60 mg/L^−1^ in ultrapure water (pH = 5.63) and in seawater (pH = 8.46). Figure 1 shows the Pb^II^ uptake from aqueous solutions of various concentrations after 24 h. Such a long incubation time was chosen in order to ensure that equilibrium was established, as determined by the plateau in the uptake-versus-time curve. For the Pb^II^ uptake from ultrapure water solutions (Figure 1A), the adsorption capacity of the X-Ca-alginate aerogel beads increased from 0.02 to 29 mg/g^−1^ over the concentration range of 0.01 to 50 mg/L^−1^ and then remained constant. The Pb^II^ uptake was quantitative or almost quantitative for initial concentrations up to 5 mg/L^−1^. More specifically, no residual Pb^II^ could be detected in solutions with initial concentrations of 0.01 and 0.1 mg/L^−1^, while >97% of Pb^II^ was adsorbed for initial concentrations in the 1–5 mg/L^−1^ range, in which case 75% of Pb^II^ was adsorbed in 2.5 h and 90% of Pb^II^ was adsorbed in 6 h (Supplementary Materials Figure S5). The maximum Pb^II^ uptake observed (29 mg Pb^II^ or 0.14 m/mol Pb^II^ per g of aerogel) corresponds to the replacement of only one-third of the calcium initially contained in the X-Ca-alginate aerogels (19 mg Ca^II^ or 0.47 m/mol Ca^II^ per g of X-Ca-alginate aerogel).

**Figure 2:**
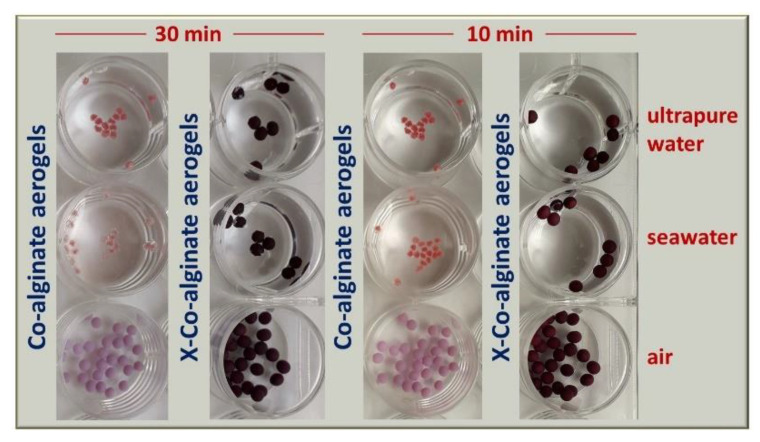
Optical photographs comparing the stability of native M-alginate hydrogels and aerogels and of X-M-alginate aerogels in tap water, seawater, and air. Co^II^ (instead of Ca^II^) was used as the gelation cation for alginate because it provides pink materials that increase the imaging contrast.

To examine whether X-Ca-alginate aerogels can be used for the decontamination of aquatic environments, the adsorption of Pb^II^ from seawater collected from an unpolluted marine area was also tested (Figure 1C). Pertinent studies in seawater are particularly scarce in the literature [10,11,21,51]. Several organic and inorganic compounds present in natural waters fundamentally affect the efficiency of Pb^II^ adsorption. Adsorption measurements were conducted according to the experimental protocol followed in the case of ultrapure water tests. The Pb^II^ uptake was quantitative for the lowest concentration (i.e., 0.01 mg/L^−1^). The maximum adsorption capacity (13 mg Pb^II^ or 0.06 m/mol Pb^II^ per g of aerogel) was about half of that from ultrapure water solutions. This finding is not surprising, because the higher ionic strength of seawater is expected to affect the adsorption capacity [51]: the presence of several metal ions at high concentrations (mostly Na^+^, K^+^, Mg^2+^, Ca^2+^) competes with Pb^II^ toward complexation to the alginate network. In addition, native alginate aerogels cannot be used in seawater; they shrink within minutes (Figure 2), as Na^I^ ions replace the Ca^II^ ions, which maintain the alginate network. On the other hand, owing to the fact that in crosslinked aerogels the alginate network is reinforced and held in place by PUA, X-Ca-alginate aerogels withstand shrinkage/disintegration and beads are stable in seawater for at least a week.

The data points of the adsorption experiments were fitted with the Langmuir (Equation (1)) [52] and the Freundlich (Equation (2)) [53] sorption isotherm models. *Q*_eq_ is the Pb^II^ uptake (in mg/g^−1^) at equilibrium, *C*_eq_ is the Pb^II^ concentration in the solution at equilibrium (in mg/L^−1^), *Q*_max_ is the adsorption capacity (in mg/g^−1^), *K*_L_ is the Langmuir equilibrium constant (in L/mg^−1^), *K*_F_ is the Freundlich equilibrium constant, and *n* is the empirical adsorption intensity of the Freundlich model that represents a measure of nonlinearity. The corresponding plots and calculated parameters are shown in Figure 1B,D and Supplementary Materials Figure S6. As it is already obvious from the plots of the Pb^II^ uptake vs. the initial Pb^II^ concentration (Figure 1A,C), the adsorption follows different mechanisms in ultrapure water and seawater. The Langmuir model gave the best fit of the experimental data (R^2^ = 0.95; Figure 1B) in ultrapure water. A maximum adsorption capacity of 88 mg/g^−1^ was calculated from that model, which is close to the adsorption expected if all Ca^II^ ions were replaced by Pb^II^ (97 mg/g^−1^). That maximum adsorption capacity could not be achieved experimentally, because of the low value of the calculated equilibrium constant (*K*_L_ = 0.13 L/mg^−1^). The Freundlich model was also fitted (Supplementary Materials Figure S6), and the value of *n* (calculated from the linear part of the plot) was found to be equal to 1.9, indicating that the adsorption was favored (*n* > 1). The Freundlich model fitted the experimental data (R^2^ = 0.99; Figure 1D) better in seawater. The value of *n* calculated from the Freundlich model was equal to 1.2, in agreement with the observed linear adsorption isotherm.

(1) $$Q_{\mathrm{eq}}\;=\;\frac{Q_{\max}\;K_{L}\;C_{\mathrm{eq}}}{1\;+\;K_{L}\;C_{\mathrm{eq}}}$$

(2) $$Q_{\mathrm{eq}}\;=\;K_{F}\;C_{\mathrm{eq}}{}^{1/n}$$

**Figure 3:**
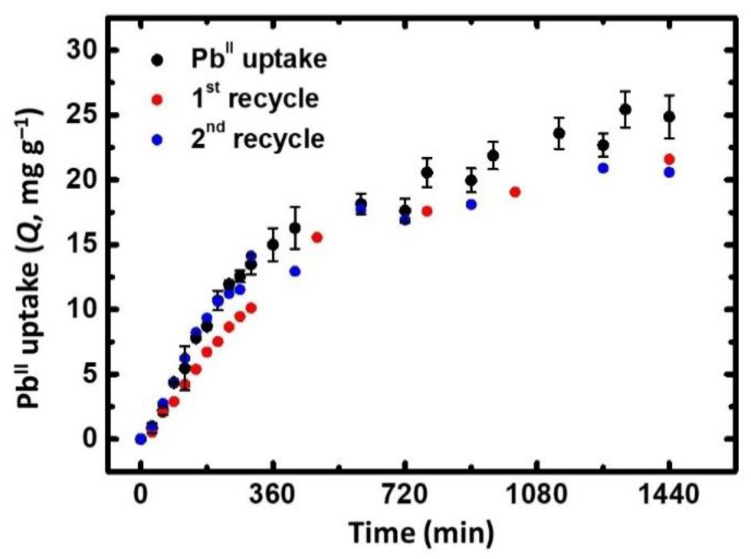
Black dots: Pb^II^ uptake from ultrapure water solutions by X-Ca-alginate aerogel beads versus time. Red dots: 1st recycle. Blue dots: 2nd recycle. Initial Pb^II^ concentrations: 40 mg/L^−1^.

Time-resolved adsorption experiments were performed under the same conditions as the batch adsorption tests. As seen in Figure 3 (black dots), X-alginate beads reached 85% of their maximum Pb^II^ adsorption capacity after 13 h of agitation at 40 mg/L^−1^ of the initial Pb^II^ concentration. Interestingly, X-Ca-alginate aerogels could be recycled twice without significant loss of their adsorption capacity (Figure 3, compare the red and blue dots). This was accomplished by treating previously used X-Ca-alginate beads with a solution of Na_2_EDTA, washing them thoroughly with distilled water, and reusing them.

### 2.3. Adsorption of Organic Solvents and Oils and Separation of Organic Solvents and Oil from Seawater

**Figure 4:**
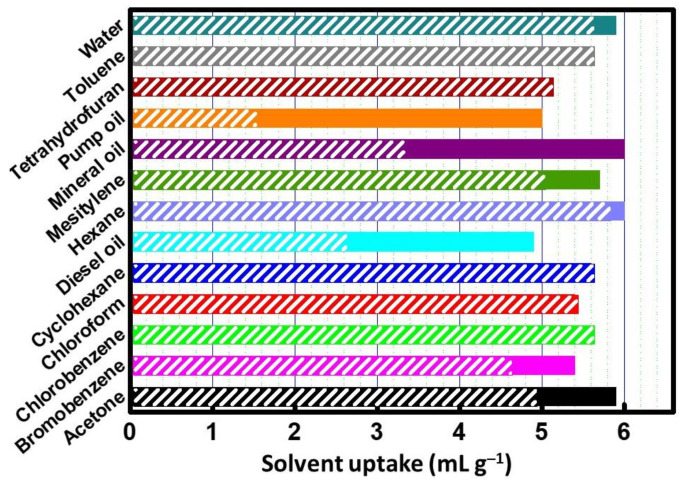
Solvent adsorption by X-Ca-alginate aerogel beads at 10 min (shaded length of each column) and at the time of maximum adsorption, i.e., 1 h for all solvents in Table 1, 1.5 h for diesel oil, 2.5 h for mineral oil, and 4 h for pump oil (full length of each column).

X-Ca-alginate aerogels were also evaluated for their ability to adsorb organic solvents and also for their ability to separate organic solvents and oil from seawater. Representative examples of common aliphatic, aromatic, chlorinated, and brominated organic solvents that can comprise environmental hazards were chosen. Table 1 and Table 2 and Figure 4 summarize the results from solvent adsorption. For all solvents tested, the maximum solvent uptake was around 6 mL/g^−1^, equal to the total pore volume of the aerogel beads (6.0 mL/g^−1^; Supplementary Materials Table S1), and the process was complete (or almost complete) within 10 min. The absorption of oils (diesel, mineral, and pump oils) was not as fast as that of organic solvents; it required 1.5 h to reach its maximum value for diesel oil, 2.5 h for mineral oil, and 4 h for the more viscous pump oil (Table 2). X-alginate aerogel beads are insoluble, and they do not swell in any of the above solvents.

**Figure 5:**
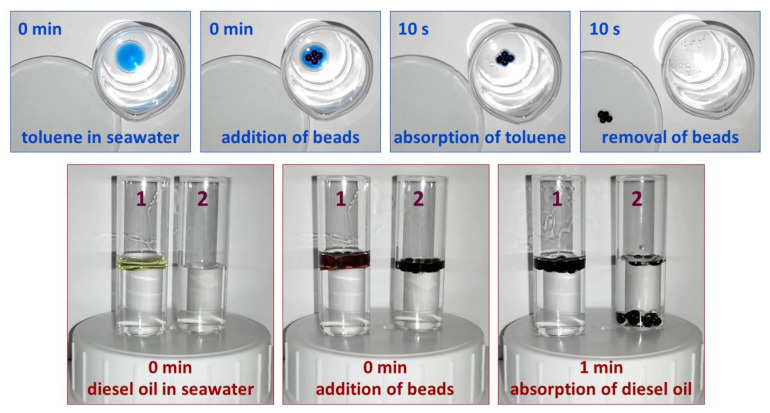
Selective uptake of toluene colored with Sudan blue (top row, view from top) and of diesel oil (bottom row, side view) from seawater using X-Ca-alginate aerogel beads. In the bottom row, vial 1 contains diesel oil and seawater and vial 2 contains only seawater. After 1 min, in vial 1, the beads absorbed diesel oil and they floated, while in vial 2, they absorbed water and they sank.

For separation of organic solvents or oil from seawater, solvent mixtures of toluene, hexane, and cyclohexane in seawater (3% *v*/*v*) were prepared and the corresponding amount of X-alginate aerogel beads was added (see Table 1). The absorption of the solvents was extremely fast and complete within 10 s. The separation of diesel oil from seawater was also studied. To expedite the adsorption of diesel oil, a larger number of beads (2×) was used compared to the amount calculated from Table 2. That accelerated the adsorption of diesel oil, and the process was complete within 1 min. Representative photographs are shown in Figure 5. In any case, the organic phase was selectively adsorbed because of the hydrophobic nature of the beads. No desorption (leak) from the beads to the aqueous phase was observed even after 3 h. As a control experiment, the absorption of the same organic solvents and diesel oil was also studied with ultrapure water instead of seawater; the absorption capacity was the same. PUA aerogels prepared from the trimer of hexamethylene diisocyanate [24] of the same density as X-Ca-alginate aerogel beads behaved similarly toward oil uptake for water. X-Ca-alginate aerogel beads performed equally well and sometimes better than several sorbents based on inorganic materials (e.g., magnesium carbonate [54] or barium sulfate [55]), natural products (e.g., rice husk ash [56]), or composite materials (e.g., Fe_3_O_4_/poly(methylmethacrylate/styrene/divinylbenzene [57]).

## 3. Conclusions

Polyurea-crosslinked Ca-alginate (X-Ca-alginate) aerogel beads (diameter: 3.3 mm) were prepared via reaction of an aromatic triisocyanate (Desmodur RE) with pre-formed Ca-alginate wet gels. They are hydrophobic nanoporous materials (90% *v*/*v* porosity), with a high BET surface area (459 m^2^/g^−1^), consisting of 54% *w*/*w* polyurea and 2% *w*/*w* calcium, and they have been tested for potential application in environmental remediation as adsorbents of metal ions and organic solvents. X-Ca-alginate aerogels selectively adsorb Pb^II^ from ultrapure water (29 mg/g^−1^; pH = 5.63). Adsorption of Pb^II^ from seawater (13 mg/g^−1^; pH = 8.46) can also be achieved, although the adsorption capacity is lower compared to solutions of ultrapure water, presumably because of the higher ionic strength of seawater. Pb^II^ replaces Ca^II^ in the alginate network and coordinates to the carboxylate groups. Pb^II^ can be removed by placing the beads in a solution of Na_2_EDTA; afterward, the beads can be used again for Pb^II^ adsorption from new contaminated water samples, without significant loss of activity for at least two cycles. X-Ca-alginate aerogels can also adsorb organic solvents and oil from seawater; the volume of the adsorbent can be as high as the total pore volume of the aerogel, and the absorption process can be complete in less than 1 min.

X-Ca-alginate aerogel beads are hydrophobic; therefore, they can be stored and deployed easily. In addition, they are stable in water and, most importantly, in seawater, while native alginate aerogels shrink within a few minutes. Future work will focus on the preparation of X-Ca-alginate aerogel beads of various diameters and with coating from different polyureas for adjustable hydrophobicity, and they will be evaluated for the decontamination of aquatic environments (e.g., seawater, river water, industrial wastewater) from a broader range of inorganic and organic pollutants.

## 4. Experimental Section

### 4.1. Materials and Methods

Sodium alginate PROTANAL LF 240 D (G/M = 0.43–0.54) was used as starting material. CaCl_2_, CoCl_2_·6H_2_O, toluene, mesitylene, and bromoethane were purchased from Sigma (Saint Louis, MO, USA). Desmodur RE (27% *w/w* triphenylmethane-4,4’,4’’-triisocyanate (TIPM) solution in ethyl acetate (EA)) was generously provided by Covestro AG (Leverkusen, Germany). MeCN (HPLC grade), chloroform, hexane, cyclohexane, and tetrahydrofuran were purchased from Fisher Scientific (Waltham, MA, USA). Acetone and Na_2_EDTA were purchased from Lach-Ner (Neratovice, Czechia). Chlorobenzene was purchased from Chem-Lab Analytical (Zedelgem, Belgium). All solvents were used as received.

Supercritical fluid (SCF) drying was carried out in an autoclave (Model E3100, Quorum Technologies, East Sussex, UK). Wet gels were placed in the autoclave at 12 °C and were covered with acetone. Liquid CO_2_ was allowed in the autoclave; acetone was drained out as it was being displaced by liquid CO_2_ (5×; 1 per 30 min). Subsequently, the autoclave temperature was raised to 45 °C, and it was maintained for 1 h. Finally, the pressure was gradually released, allowing SCF CO_2_ to escape as a gas, leaving aerogels.

Solid-state cross-polarization magic angle spinning (CPMAS) ^13^C NMR spectra were obtained with a 600 MHz Varian spectrometer (Varian, Palo Alto, CA, USA) operating at 150.80 MHz for ^13^C. Attenuated total reflection Fourier transform IR (ATR-FTIR) spectra were obtained with a Perkin Elmer Spectrum 100 Spectrometer.

N_2_-sorption measurements were performed on a Micromeritics Tristar II 3020 surface area and porosity analyzer (Micromeritics, Norcross, GA, USA). Skeletal densities (*ρ_s_*) were determined by He pycnometry using a Micromeritics AccuPyc II 1340 pycnometer (Micromeritics, Norcross, GA, USA). Bulk densities (*ρ_b_*) of the samples were calculated from their weight and natural dimensions.

The calcium content of X-Ca-alginate aerogel beads was determined with atomic emission spectrometry (AES) employing a Varian SpectrAA 200 instrument (Varian, Mulgrave, Australia), following by wet digestion of the beads with supra-pure HNO_3_ 65% (Merck, Darmstadt, Germany).

### 4.2. Synthesis of X-Ca-Alginate Aerogel Beads

X-Ca-alginate aerogel beads were prepared according to a literature procedure [41]. In brief, an aqueous solution of sodium alginate (3% *w/w*) was added dropwise, using a 25 mL burette, to a 0.2 M solution of a metal salt (CaCl_2_) under mild magnetic stirring. Spherical hydrogel alginate beads were formed instantly, and they were left to age for 24 h; after that time, they were stepwise solvent-exchanged with MeCN/H_2_O mixtures (30, 60, 90% *v/v*) and finally with MeCN (4×). Subsequently, the wet-gel beads were kept in a solution of a triisocyanate in EA/MeCN (0.75 M) for 24 h at room temperature and for 72 h at 70 °C in order to complete the crosslinking reaction. Afterward, crosslinked Ca-alginate (X-Ca-alginate) wet-gel beads were solvent-exchanged with acetone (3×) and were dried with SCF CO_2_ to the corresponding aerogels.

### 4.3. Pb^II^ Uptake from Aqueous Solutions

Accurately weighed quantities of X-Ca-alginate aerogel beads were added to plastic vials containing Pb^II^ solutions, prepared from a stock standard Pb^II^ solution (Merck) of either ultrapure water (Millipore) or seawater, at given concentrations ranging from 0.01 to 60 mg/L^−1^. The vials had been previously cleaned with supra-pure HNO_3_ 10% (Merck) and rinsed with ultrapure water of 18.2 MΩ/cm (Millipore, Bedford, MA, USA). For the preparation of all required solutions, class A volumetric glassware was used. The beads remained in each solution under continuous stirring for a given time. Subsequently, concentrations of remaining Pb^II^ in ultrapure water solutions were measured with graphite furnace atomic absorption spectrometry (GFAAS), employing a Varian SpectrAA 640Z with Zeeman background correction (Varian) (limit of detection (LOD) equal to 0.87 μg/L^−1^), for solutions with the given concentrations of 0.01 and 0.1 mg/L^−1^ and with flame atomic absorption spectrometry (FAAS), employing a Varian SpectrAA 200 instrument (Varian) (LOD equal to 0.04 mg/L^−1^), following appropriate dilution of the samples for the rest of Pb^II^ solutions. In the case of seawater solutions, corresponding direct measurements were performed electrochemically using a μAutolab type III (Eco-Chemie, Utrecht, the Netherlands) instrument connected to a three-electrode cell (663 VA Stand, Metrohm, Herisau, Switzerland) with a static mercury drop electrode (SMDE) as the working electrode. The reference electrode was a Ag/AgCl (3 M KCl) electrode, while a carbon-rod electrode served as the auxiliary electrode. Throughout the experiment, the temperature was maintained equal to 20 °C. The uptake of Pb^II^ (*Q*; mg/g^−1^) was calculated as the ratio of the mass of Pb^II^ ions adsorbed to the mass of X-Ca-alginate aerogel beads used, according to Equation (3).

(3) $$Q\;=\;\frac{\mathrm{mass}\;\mathrm{of}\;\mathrm{Pb}\;\mathrm{ions}\;\mathrm{adsorbed}}{\mathrm{mass}\;\mathrm{of}\;\mathrm{aerogel}\;\mathrm{beads}}$$

### 4.4. Solvent Uptake

Accurately weighed quantities of X-Ca-alginate aerogel beads were kept in a tube containing an organic solvent, an oil, or water under mild stirring. At selected time intervals, the beads were taken out of the tube and weighed. Afterward, the beads were re-immersed in the respective solvent, and the above steps were repeated for 1 h. The temperature was 20 °C. The solvent uptake (*q*; mL/g^−1^) was calculated as the ratio of the volume of the solvent adsorbed to the mass of the X-Ca-alginate aerogel beads used, according to Equation (4).

(4) $$q\;=\;\frac{(\mathrm{mass}\;\mathrm{of}\;\mathrm{wet}\;\mathrm{beads}\;-\;\mathrm{mass}\;\mathrm{of}\;\mathrm{aerogel}\;\mathrm{beads})/\mathrm{density}\;\mathrm{of}\;\mathrm{the}\;\mathrm{solvent}}{\mathrm{mass}\;\mathrm{of}\;\mathrm{aerogel}\;\mathrm{beads}}$$

### 4.5. Separation of Organic Solvents and Diesel Oil from Seawater

X-Ca-alginate aerogel beads were weighed and then added to a tube containing a mixture of seawater and an organic solvent or diesel oil (3% *v/v*). Sudan blue was used as a dye to visualize the adsorption of the organic solvent from the beads. The wet beads were removed after they had adsorbed the whole quantity of the solvent, and the time required for that was measured. The temperature was 20 °C.

## Figures and Tables

**Table 1:** Solvent adsorption by X-Ca-alginate aerogel beads at various time intervals.

Solvent	Solvent Uptake (mL/g^−1^)					
	10 min	20 min	30 min	40 min	50 min	60 min
Toluene	5.6	5.1	5.2	5.3	5.4	5.6
Mesitylene	5.0	5.7	5.2	5.6	5.6	5.7
Chlorobenzene	5.6	5.5	5.5	5.5	5.4	5.6
Chloroform	5.4	5.5	5.3	5.3	5.2	5.4
Bromoethane	4.6	5.3	5.2	5.0	5.2	5.4
Hexane	5.8	6.1	5.9	6.0	6.2	6.0
Cyclohexane	5.6	5.1	5.2	5.3	5.5	5.6
Tetrahydrofuran	5.1	4.8	5.0	4.9	5.1	5.1
Acetone	4.9	4.9	5.9	5.6	5.8	5.9
Water	5.6	5.6	5.5	5.8	5.8	5.9

**Table 2:** Oil adsorption by X-Ca-alginate aerogel beads at various time intervals.

Oil	Solvent Uptake (mL/g^−1^)									
	10 min	30 min	60 min	90 min	120 min	150 min	180 min	240 min	300 min	360 min
Diesel oil	2.6	3.6	4.3	4.8	4.7	4.7	4.8	4.7	4.9	4.9
Mineral oil	3.3	4.5	5.6	5.5	5.6	5.9	6.0	5.9	5.8	6.1
Pump oil	1.4	1.7	2.7	2.8	2.7	3.0	3.8	5.0	4.9	4.9

## Data Availability

The data presented in this study are available in the article and in the supplementary materials section.

## Supplementary Materials

The following are available online at https://www.mdpi.com/2310-2861/7/1/27/s1: Figure S1: Optical photograph and size distribution of X-Ca-alginate aerogel beads (diameters measured with ImageJ; histogram calculated using OriginPro 9.0). Mean diameter and sample size (N) are shown on the figure. Figure S2: ATR-FTIR spectra of X-Ca-alginate aerogel beads, as indicated. The characteristic peaks for the Ca-alginate skeleton are noted with blue, and the ones for polyurea (PUA) are noted with purple. Figure S3: ^13^C CPMAS NMR spectra of X-Ca-alginate aerogel beads. Figure S4: N_2_-sorption diagram of crosslinked X-Ca-alginate aerogel beads. Inset shows pore size distribution by the BJH method. Figure S5: Pb^II^ uptake from ultrapure water solutions by X-Ca-alginate aerogel beads versus time. Initial Pb^II^ concentrations: 0.01 (**A**), 0.1 (**B**), and 1 (**C**) mg/L^−1^. Figure S6: Freundlich isotherm for Pb^II^ uptake from ultrapure water solutions by X-Ca-alginate aerogel beads. *Q*_eq_: Pb^II^ uptake at equilibrium. *C*_eq_: concentration of Pb^II^ in the solution at equilibrium. Table S1: Selected material properties of X-Ca-alginate aerogel beads.
