# Supplementary material for: Evaluation of Polyurea-Crosslinked Alginate Aerogels for Seawater Decontamination
Source: Gels. 2021 Mar 4;7(1):27. doi: 10.3390/gels7010027 (PMC8005931; doi:10.3390/gels7010027)
Supplement: Supplementary file 1 [file gels-07-00027-s001.pdf]

Evaluation of Polyurea-Crosslinked Alginate Aerogels for Seawater Decontamination

Patrina Paraskevopoulou [1,*], Grigorios Raptopoulos [1], Faidra Leontaridou [1], Maria Papastergiou [1], Aikaterini Sakellari [2] and Sotirios Karavoltsos [2,*]

[1] Inorganic Chemistry Laboratory, Department of Chemistry, National and Kapodistrian University of Athens, Panepistimiopolis Zografou, 15771 Athens, Greece; grigorisrap@chem.uoa.gr (G.R.); faidraleo@chem.uoa.gr (F.L.); mapapast@chem.uoa.gr (M.P.)
[2] Laboratory of Environmental Chemistry, Department of Chemistry, National and Kapodistrian University of Athens, Panepistimiopolis Zografou, 15784 Athens, Greece; esakel@chem.uoa.gr (A.S.)
* Correspondence: paraskevopoulou@chem.uoa.gr; Tel.: +30-210-727-4381(P.P.); skarav@chem.uoa.gr; Tel.: +30-210-727-4269 (S.K.)

Table of contents

Figure S1. Optical photograph and size distribution of X-Ca-alginate aerogel beads (diameters measured with ImageJ; histogram calculated using OriginPro 9.0). Mean diameter and sample size (N) are shown on the Figure. ... 2
Figure S2. ATR-FTIR spectra of X-Ca-alginate aerogel beads, as indicated. The characteristic peaks for the Ca-alginate skeleton are noted with blue and the ones for polyurea (PUA) are noted with purple. 2
Figure S3. ^{13}C CPMAS NMR spectra of X-Ca-alginate aerogel beads. 3
Figure S4. N_2-sorption diagram of crosslinked X-Ca-alginate aerogel beads. Inset shows pore size distribution by the BJH method. 3
Figure S5. Pb^{II} uptake from ultrapure water solutions by X-Ca-alginate aerogel beads versus time. Initial Pb^{II} concentrations: 0.01 (A), 0.1 (B) and 1 (C) mg L^{-1}. 4
Figure S6. Freundlich isotherm for Pb^{II} uptake from ultrapure water solutions by X-Ca-alginate aerogel beads. Q_{eq}: Pb^{II} uptake at equilibrium. C_{eq}: concentration of Pb^{II} in the solution at equilibrium. 4

Table S1. Selected material properties of X-Ca-alginate aerogel beads. 5

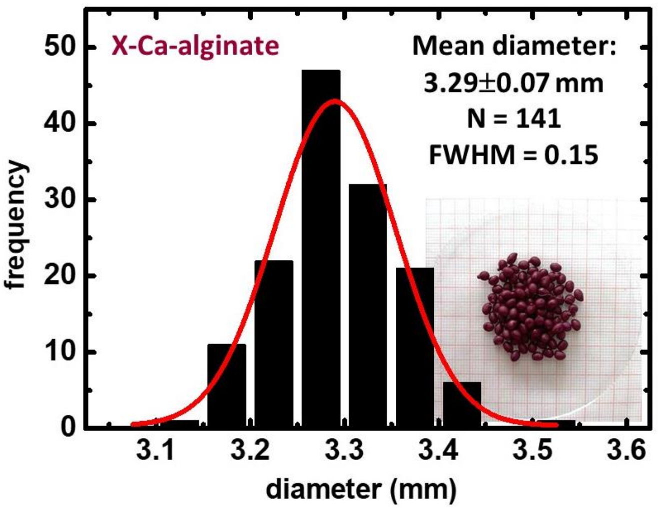

Figure S1. Optical photograph and size distribution of X-Ca-alginate aerogel beads (diameters measured with ImageJ; histogram calculated using OriginPro 9.0). Mean diameter and sample size (N) are shown on the Figure.

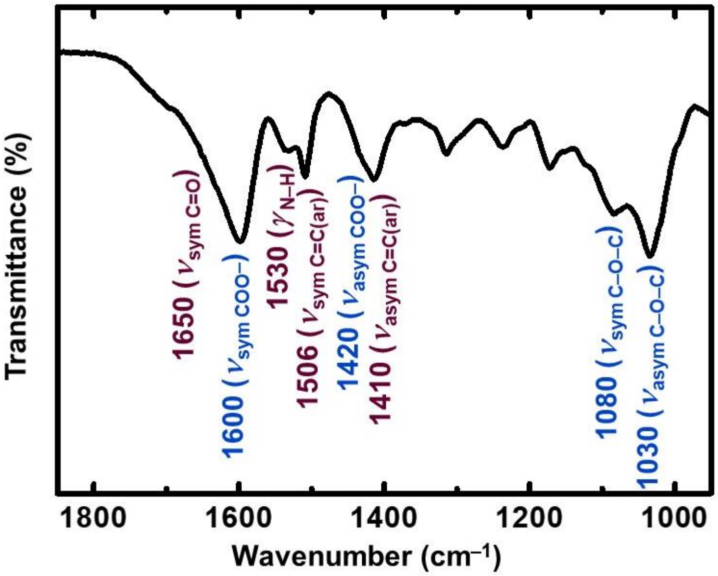

Figure S2. ATR-FTIR spectra of X-Ca-alginate aerogel beads, as indicated. The characteristic peaks for the Ca-alginate skeleton are noted with blue and the ones for polyurea (PUA) are noted with purple.

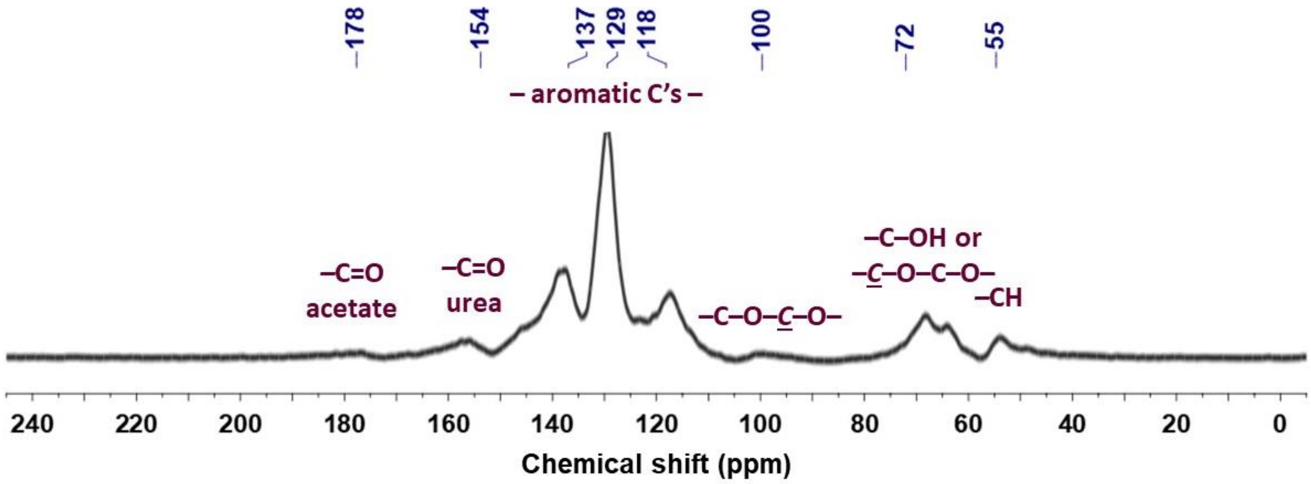

Figure S3. ^{13}C CPMAS NMR spectra of X-Ca-alginate aerogel beads.

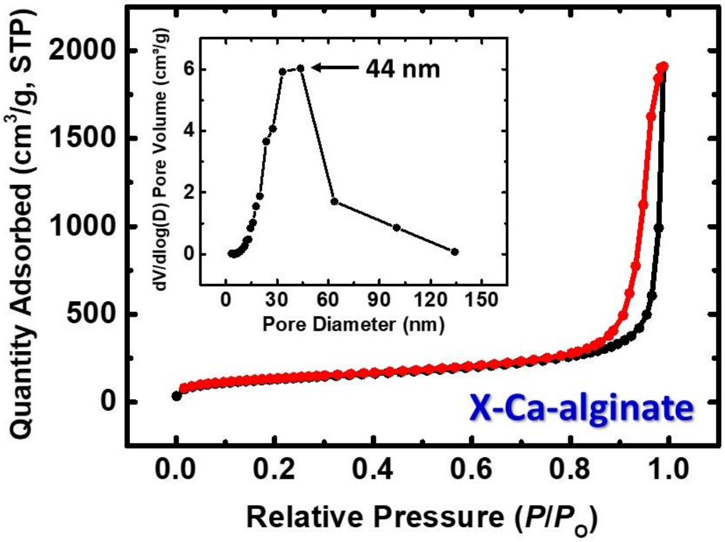

Figure S4. N_2-sorption diagram of crosslinked X-Ca-alginate aerogel beads. Inset shows pore size distribution by the BJH method.

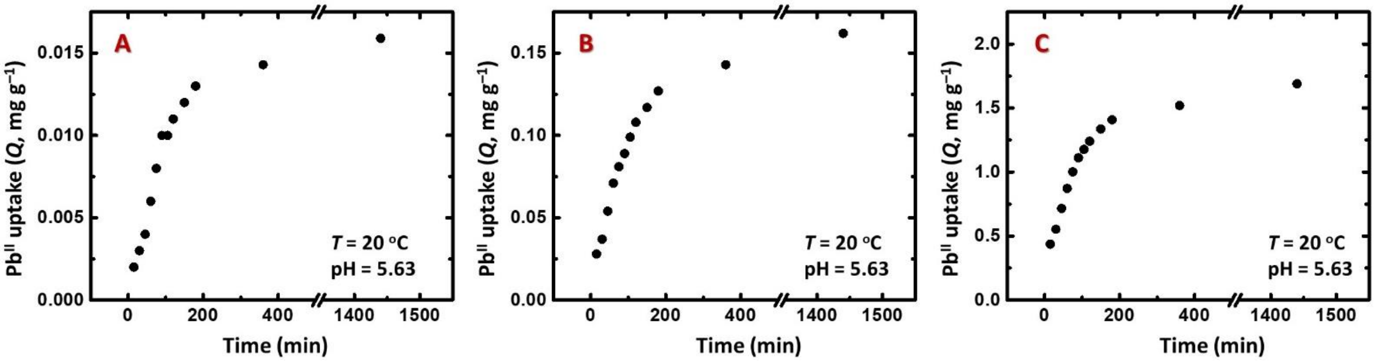

Figure S5. Pb^{II} uptake from ultrapure water solutions by X-Ca-alginate aerogel beads versus time. Initial Pb^{II} concentrations: 0.01 (A), 0.1 (B) and 1 (C) mg L^{-1}.

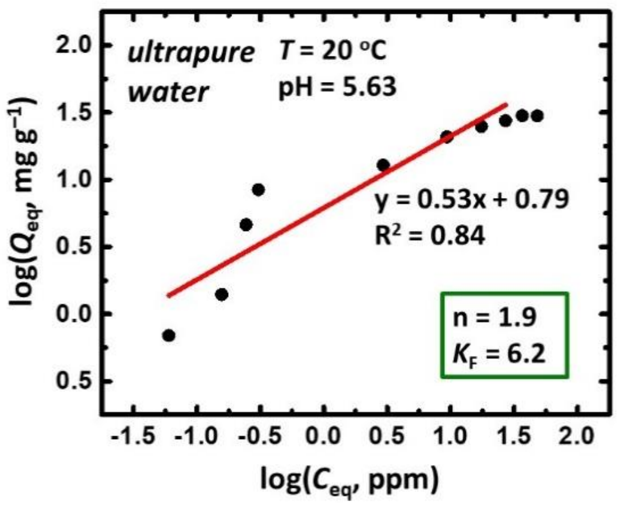

Figure S6. Freundlich isotherm for Pb^{II} uptake from ultrapure water solutions by X-Ca-alginate aerogel beads. Q_{eq}: Pb^{II} uptake at equilibrium. C_{eq}: concentration of Pb^{II} in the solution at equilibrium.

Table S1. Selected material properties of X-Ca-alginate aerogel beads.

Sample [a]	Bulk density ρ_b (g cm^{-3})	Skeletal density ρ_s (g cm^{-3})	Porosity [b] Π (% v/v)	BET surf. area σ (m^2 g^{-1}) [micropore surf. area] [c]	V_{Total} [d] ($V_{1.7\text{-}300nm}$) [e] (cm^3 g^{-1})	Av. pore diam. [f] (4V_{Total}/σ) (nm)	Particle radius [g] r (nm)
**X-Ca-alginate aerogel beads**	0.150±0.009	1.485±0.005	90	459 [28]	6.0 (2.9)	25 (50)	4.4 (4.7)

[a] The concentration of the sodium alginate solution was 3% w/w. [b] Porosity calculated according to the formula: $(\rho_s-\rho_b)/\rho_s$, where ρ_s: skeletal density and ρ_b: bulk density. [c] Micropore surface area *via* *t*-plot analysis, according to the Harkins and Jura model. [d] Total pore volume calculated according to formula: $1/\rho_b-1/\rho_s$. [e] Cumulative volume of pores between 1.7 and 300 nm from N_2-sorption data and the BJH desorption method. [f] Calculated by the 4 V/σ method; V was set equal to the maximum volume of N_2 adsorbed along the isotherm as $P/P_o \rightarrow 1.0$. For the number in parentheses, V was set equal to V_{Total} from the previous column. [g] Particle radius calculated by the formula: $r = 3/(\rho_s \times \sigma)$, where σ: BET surface area. For the number in parentheses, σ was set equal to the external surface area, σ_{ext}, calculated from the BET surface area minus the micropore surface area.
